# High-Fat Diet and Short-Term Unpredictable Stress Increase Long-Chain Ceramides Without Enhancing Behavioral Despair

**DOI:** 10.3389/fmolb.2022.859760

**Published:** 2022-05-04

**Authors:** Lubriel Sambolín-Escobales, Lizmarie Tirado-Castro, Cristina Suarez, Dariangelly Pacheco-Cruz, Wilfred Fonseca-Ferrer, Pragney Deme, Norman Haughey, Gladys Chompre, James T. Porter

**Affiliations:** ^1^ Division of Pharmacology, Basic Sciences Department, Ponce Research Institute, Ponce Health Sciences University, Ponce, Puerto Rico; ^2^ Biology and Biotechnology Department, Pontifical Catholic University of Puerto Rico, Ponce, Puerto Rico; ^3^ Biology Department, University of Puerto Rico, Ponce, Puerto Rico; ^4^ Department of Neurology, John Hopkins University School of Medicine, Baltimore, MD, United States

**Keywords:** ceramides, depressive-like behavior, inflammation, unpredictable stress, anhedonia, sex-dependent

## Abstract

Clinical and preclinical studies suggest that increases in long-chain ceramides in blood may contribute to the development of depressive-like behavior. However, which factors contribute to these increases and whether the increases are sufficient to induce depressive-like behaviors is unclear. To begin to address this issue, we examined the effects of high fat diet (HFD) and short-term unpredictable (STU) stress on long-chain ceramides in the serum of male and female rats. We found that brief exposure to HFD or unpredictable stress was sufficient to induce selective increases in the serum concentrations of long-chain ceramides, associated with depression in people. Furthermore, combined exposure to HFD and unpredictable stress caused a synergistic increase in C16:0, C16:1, and C18:0 ceramides in both sexes and C18:1 and C24:1 in males. However, the increased peripheral long-chain ceramides were not associated with increases in depressive-like behaviors suggesting that increases in serum long-chain ceramides may not be associated with the development of depressive-like behaviors in rodents.

## Introduction

Ceramides are bioactive lipids that orchestrate various cellular processes depending on their structure, specificity, and cellular location (Ogretmen and Hannun., 2004; Won et al., 2004; Gracia-Garcia et al., 2011; Hannun and Obeid, 2011). Increased levels of long-chain ceramides, C16:0, C18:0, C20:0, C22:0, and C24:0, have been found in the plasma of patients with major depressive disorder (MDD) suggesting that higher levels of these ceramides may contribute to the development of MDD (Gracia-Garcia et al., 2011; Mielke et al., 2013; Brunkhorst-Kanaan et al., 2019). Fluorescent-labeled C6 ceramide appeared in the brain 30 min after intravenous administration to adult male rats (Zimmermann et al., 2001), suggesting that ceramides in the serum may cross the blood-brain-barrier. Moreover, localized infusions of C16:0 ceramides into dorsal hippocampus of male mice induced anhedonia-like behavior, suggesting that increased ceramides in the brain are sufficient to induce depressive-like symptoms (Gulbins et al., 2013).

In rodent models, high fat diet (HFD) can lead to the accumulation of peripheral ceramides. Exposure to 13–16 weeks of HFD increases C16:0, C18:0, C20:0, C22:0 and C24:0 ceramides in the serum of male mice (Shah et al., 2008; Boini et al., 2010). Furthermore, 16 weeks of HFD also induces anhedonia-like behavior seen as reduced sucrose preference (Dutheil et al., 2016) and increased behavioral despair in the forced swim test in adult male rats (Aslani et al., 2015). Taken together, these studies suggest that HFD could increase peripheral long-chain ceramides to induce depressive-like behaviors. However, since ceramides and behavior were not assessed together, the relationship between ceramide changes and depressive-like behaviors is unknown. In addition, since only male rodents were evaluated in previous experiments, it is unclear whether female rodents show similar changes in ceramides and behavior in response to HFD.

Another factor that can increase peripheral ceramides is unpredictable stress (Wang et al., 2021) which also induces depressive-like behaviors after several weeks in adult male rodents (Liu et al., 2013; Jain et al., 2016; Xue et al., 2020). In our study, we aimed to determine whether unpredictable stress affects specific long-chain ceramide concentrations or alters the effects of HFD on serum ceramides in male and female rats. To avoid ceiling effects and increase the probability of identifying synergistic effects, we chose to combine modest duration HFD with 4 days of unpredictable stress (STU stress) which is below the 7–10 days of unpredictable stress needed to induce depressive-like phenotypes in rodents (Zurita et al., 2000; Maslova et al., 2002; Batschauer et al., 2020; Meng et al., 2020; Fang et al., 2021). We found that although exposing male and female rats to HFD and/or STU stress increased certain peripheral long-chain ceramide levels, the increases did not correlate with changes in anhedonia-like behavior or behavioral despair.

## Materials and Methods

### Animals

All animals used for behavioral experiments and tissue collection for molecular analysis were treated according to the legal and ethics requirements of the Institutional Animal Care and Use Committee (IACUC). Forty-seven male and forty-eight female 21-day-old Sprague Dawley rats were obtained from the Animal House facilities of Ponce Health Sciences University and maintained in standard laboratory conditions (12 h light/dark cycle, 25°C), with food and water provided *ad libithum*.

### High and Low-Fat Diets

**FIGURE 1:**
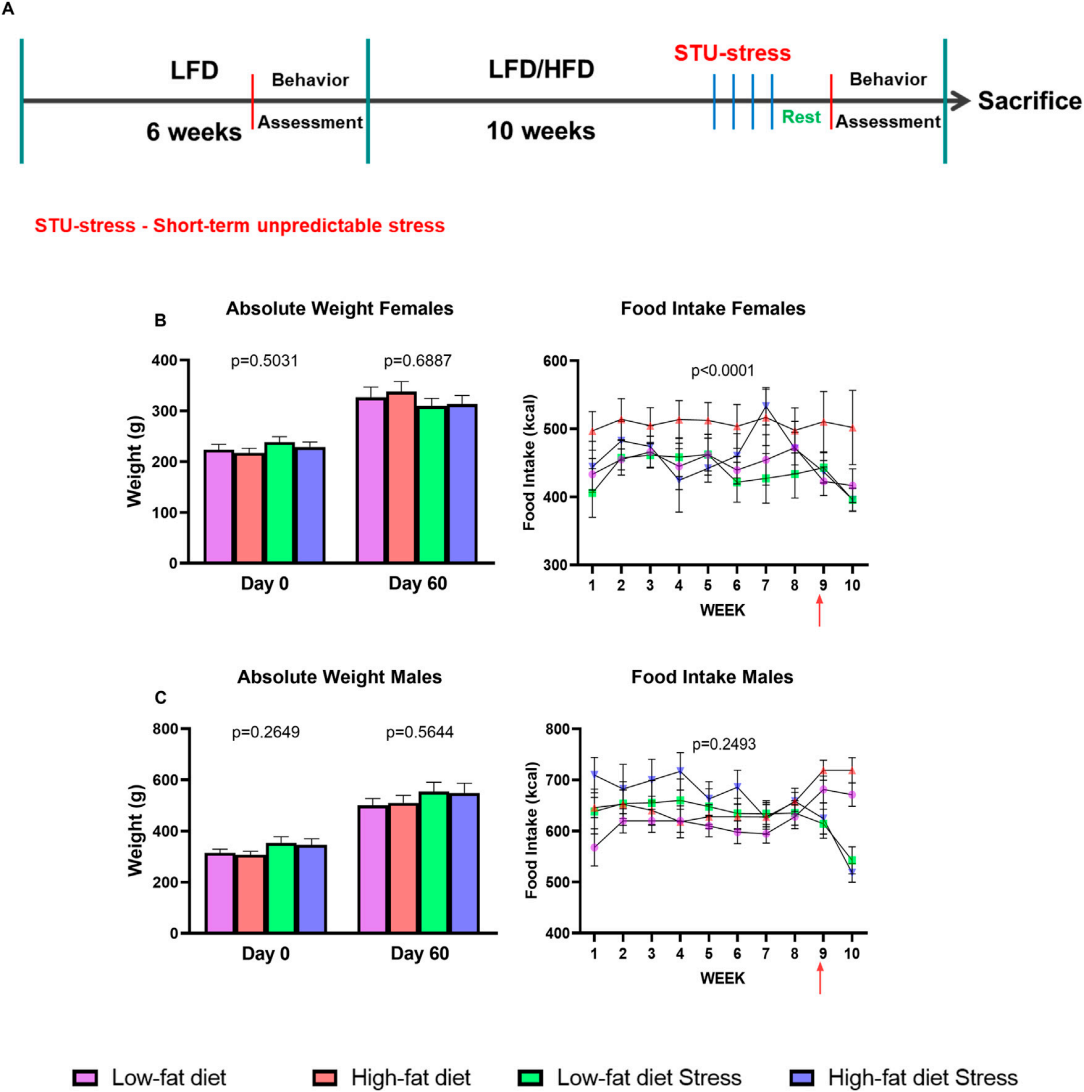
Experimental timeline and effect of HFD and/or STU stress on absolute weight and food intake**. (A)** Timeline of the exposure of male and female rats to LFD, HFD, and STU stress. **(B)** Absolute weight and food intake of female rats. Red arrow on food intake graph indicates the inclusion of the STU stress paradigm. **(C)** Absolute weight and food intake of male rats. Data represented as mean ± SEM. Red arrow on food intake graph indicates the inclusion of the STU stress paradigm. Sample size for female rats: LFD = 12, HFD = 12, LFDS = 12, and HFDS = 12. Sample size for male rats: LFD = 12, HFD = 12, LFDS = 12, and HFDS = 11. Statistic used, One-way ANOVA with Tukey’s multiple comparison test for absolute weight analysis. One-way ANOVA with repeated measures for food intake analysis.

The male and female rats were distributed in four different cohorts and arbitrarily assigned to one of the following groups: the low-fat diet (LFD) group for 8 weeks (*n* = 9) or 10 weeks (*n* = 3), the HFD group for 8 weeks (*n* = 9) or 10 weeks (*n* = 3), the low-fat diet + stress group (LFDS) for 10 weeks (*n* = 12), or the high-fat + stress group (HFDS) for 10 weeks (*n* = 12). The initial LFD and HFD groups were done for 8 weeks. Later groups were extended by 2 weeks. The LFD and LFDS groups received a diet containing 10% kcal from fat (Research Diet, New Brunswick NJ, Cat. No. D12451) and the HFD and HFDS groups received 60% kcal from fat (Research Diet, New Brunswick NJ, Cat. No. D12492). During the first 6 weeks, the rats were housed three rats per cage. The food was weighed weekly, and an average consumption was calculated for each rat. On day 40, a sucrose grooming test (SGT) was performed to measure anhedonia-like behavior before the diet switch. On day 41, a 15-min forced swim test (FST) was performed to acclimate rats to the task, and on day 42, a 5-min FST was given to all rats to evaluate despair-like behavior before the diet switch. After setting the behavioral baseline, male and female rats were either switched to the HFD or maintained on the LFD for a period of 8–10 weeks as mentioned previously, during which the rats were single housed. During the last 3 days of the protocol, the SGT, 15-min FST and 5-min FST were performed, respectively, to determine changes in behavior. The rats were sacrificed, and the brain and peripheral organs were extracted. Half of the animals were subjected to the short-term unpredictable stress protocol explained in the section below. Figure 1A shows the experimental timeline for this study.

### Short-Term Unpredictable Stress

The STU stress paradigm was performed during four consecutive days, starting 8 days before sacrifice. This paradigm consists of a variety of stressors applied pseudorandomly over four consecutive days along with either the low-fat or high-fat diet. The stressors were applied as shown in Table 1.

**TABLE 1:** Schedule of STU stress paradigm. Stressors were applied pseudorandomly from day 108–111.

Day	Stressor	Duration	Time
Day 1	Restraint Stress	2 h	9:00 a.m.–11 a.m.
	Lights off during daytime	3 h	1:30 p.m.–4:30 p.m.
Day 2	Wet bedding in home cage	12 h	7:00 a.m.–7:00 p.m.
	Water deprivation	12 h	7:00 p.m.–7:00 a.m.
Day 3	Lights off during daytime	3 h	12:00 p.m.–3:00 p.m.
	Lights on during nighttime	12 h	7:00 p.m.–7:00 a.m.
Day 4	Lights off during daytime	3 h	7:00 a.m.–10:00 a.m.
	Restraint stress	2 h	3:00 p.m.–5:00 p.m.

### Behavioral Tests

#### Sucrose Grooming Test

A rat was placed in a clean cage and a 10% sucrose solution was sprayed onto the dorsal coat of each rat with an atomizer spray to induce grooming behavior. The total time each animal spent grooming the face, extremities, ventral, and dorsal areas was measured from a 5-min video. Between subjects, the cage was cleaned with alcohol and wiped dry.

#### Forced Swim Test

The FST took place over 2 days. The first day, a rat was placed into a (15.75″ in height and 11.8″ in diameter) vertical cylinder and forced to swim for 15 min for acclimation to the task. The next day, the rat was placed back into the cylinder for 5 min and the time the rat spent immobile, struggling and swimming was recorded to video. Immobility is when the rat paws are practically still and only moving enough to keep the head above water level. Swimming is when the rats move the posterior and anterior paws to stay afloat while struggling is considered when the anterior extremities of the rat are above the water level. After each subject, the water was changed. The rats were towel dried and placed in a warm container to completely dry, before returning them to their home cage. This test evaluates passive (immobility) versus active (struggling) responses to stress.

### Lipid Extraction

After sacrifice, blood was drawn from the heart of the rat for analysis of ceramides in serum. The blood was centrifuged for 3 min at full speed to obtain serum which was stored at −80°C. For LC-ESI-MS/MS analysis, lipids were extracted from serum samples using a modified Bligh and Dyer method. Briefly, internal standards for ceramide (d18:1/12:0) (Avanti Polar Lipids, Alabaster, AL, United States) were added to the extraction solvent at a concentration of 1.3 μg/ml (Haughey et al., 2004; Bandaru et al., 2007). After the clear phase separation, organic layers containing crude lipid extracts were collected and dried in a nitrogen evaporator (Organomation, Berlin, MA, United States) and stored at −80°C. Dried extracts were resuspended in pure methanol prior to analysis.

### LC-ESI-MS/MS Analyses of Ceramides

Ceramides from the serum samples were separated on a C18 reverse-phase column (2.6 µm, 50 mm × 2.1 mm) with an ULTRA HPLC In-Line Filter (0.5 µm Depth Filter x 0.004 in ID) (Phenomenex, Torrance, CA, United States) using a Shimadzu ultra-fast liquid chromatography (UFLC) system (Shimadzu, Nakagyo-ku, Kyoto, Japan) coupled to a hybrid triple quadrupole LIT (linear ion trap) mass spectrometer 4000 QTRAP system equipped with Turbo Ion Spray (SCIEX, Foster City, CA, United States). Electrospray Ionization (ESI, +ve) was used to ionize these lipid species and individual ceramide species were detected by multiple reaction monitoring mode. Mass spectrometry (MS) conditions and HPLC parameters were similar to those described in previous studies (Haughey et al., 2004; Mielke et al., 2015). To monitor the quality of analysis over the run of samples, quality control samples were injected in every 10 injections. Eight-point calibration curves (0.1–1,000 ng/ml) were constructed by plotting area under the curve for each ceramide calibration standard d18:1/C16:0, d18:1/C18:0, d18:1/C20:0, d18:1/C22:0, and d18:1/C24:0 (Avanti polar lipids, Alabaster, AL, United States). Correlation coefficients for standard curves were >0.999. Ceramide concentrations were calculated by fitting the identified ceramides species to these standard curves based on acyl chain length. Instrument control and data acquisition were performed by using Analyst (version 1.4.2, SCIEX Inc. Thornhill, ON, Canada) and data analysis were completed using MultiQuant software (version 2.0, SCIEX, Thornhill, ON, Canada).

### Enzyme-Linked Immunosorbent Assay

Blood drawn from cardiac puncture was centrifuged for 3 min at full speed to obtain serum for the analysis of inflammatory cytokines using Quantikine ELISA Kit from R&D Systems (TNF-α, Cat No. RTA00; and IL-6, Cat No. R6000B). Total protein concentration of serum samples was determined using the Pierce Bicinchoninic Acid Kit (Cat No.23225, Thermo Fisher). Then, dilutions of serum samples were determined following the manufacturer’s protocols for every specific ELISA Kit. Protein concentration of samples were obtained based on the standard curve from manufacturers.

## Statistics

All data is reported as the mean ± SEM. To control for any baseline differences, we expressed behavioral changes as a fold change from each rat’s behavior prior to the diet switch. Ordinary one-way ANOVA with Tukey’s multiple comparison test was used to compare among groups within each sex in the ceramide, behavioral, ELISA, and absolute weight analysis. A one-way ANOVA with repeated measures was used to analyze food consumption. All analyses were performed using GraphPad Prism 9.2.0.

## Results

### High Fat Diet With or Without Short-Term Unpredictable Stress did Not Increase Absolute Weight Gain or Food Intake

Based on evidence that HFD and stress are each associated with increases in peripheral ceramides (Shah et al., 2008; Longato et al., 2012; Zabielski et al., 2019; Wang et al., 2021) and depressive-like behaviors (Abildgaard et al., 2011; Dutheil et al., 2016; Almeida-Suhett et al., 2017; Arcego et al., 2017), we sought to determine if adding a short-term unpredictable stress to animals on a HFD would accentuate the effects of HFD on serum long-chain ceramides. All animals were initially fed a LFD for 6 weeks and then we assessed baseline anhedonia-like and despair-like behaviors in all animals to better control for individual differences in behavior. Next, rats were either switched to a HFD or maintained on the LFD for an additional 8–10 weeks. Eight days before sacrifice, one HFD and one LFD group of rats from each sex were exposed to 4 days of STU stress or maintained in their home cages as a control (Figure 1A). Exposure to HFD and/or STU stress did not alter weight gain in male or females (Figure 1B). In addition, caloric intake was unaffected by HFD or STU stress in females but was reduced by STU stress in males compared to LFD-exposed male rats (Figure 1C). These findings suggest that the rats were not obese after exposure to 8–10 weeks of the HFD.

### High Fat Diet Alone Increased Despair-Like Behavior in Both Sexes

**FIGURE 2:**
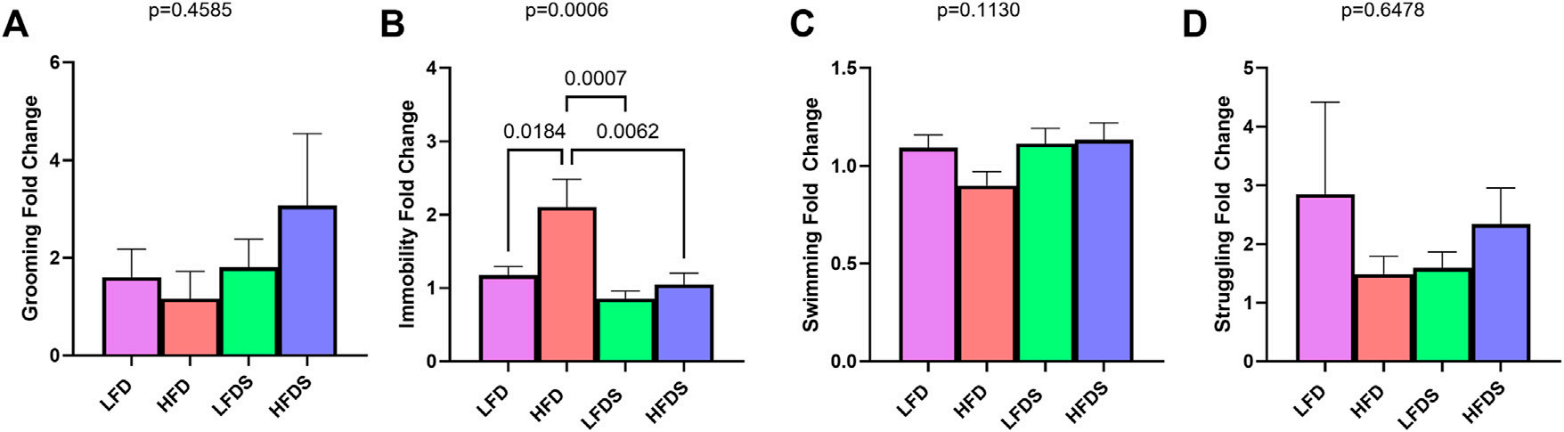
Assessment of anhedonia-like and despair-like behavior after HFD and/or STU stress. **(A)** Grooming activity in SGT in all groups. **(B–D)** Immobility, swimming, and struggling from FST. Data represented as mean ± SEM. Sample size for rats: LFD = 24, HFD = 24, LFDS (low-fat diet Stress) = 24, and HFDS (high-fat diet Stress) = 23. Statistic used, One-way ANOVA with Tukey’s multiple comparison test.

To determine whether the switch to HFD and/or the addition of STU stress altered the anhedonia-like or despair-like behavior of the rats, we calculated the fold change of each rat’s behavior from before to after the diet switch (Figure 2). The behavioral results of males and females are combined. Compared to the baseline activity, grooming time did not vary with the HFD alone, STU stress alone, or the combination of HFD + STU stress [Figure 2A, F (3.91) = 0.8724, *p* = 0.4585]. In contrast, rats did show differences in immobility in the FST [Figure 2B, F (3, 91) = 6.364, *p* = 0.0006]. The switch to HFD increased immobility (*p* = 0.0184) compared to animals fed the LFD. The inclusion of STU stress alone did not affect immobility compared to the LFD alone group (*p* = 0.7312). However, adding STU stress to the HFD reduced the immobility compared to HFD alone (*p* = 0.0062). All groups showed similar swimming time [Figure 2C, F (3, 91) = 2.046, *p* = 0.1130] and struggling time [Figure 2D, F (3, 91) = 0.5524, *p* = 0.6478]. These results suggest that the addition of the brief unpredictable stress may have reversed the increase in behavioral despair induced by the HFD.

### High Fat Diet and/or Short-Term Unpredictable Stress Increased Particular Long-Chain Ceramides in Blood Serum

**FIGURE 3:**
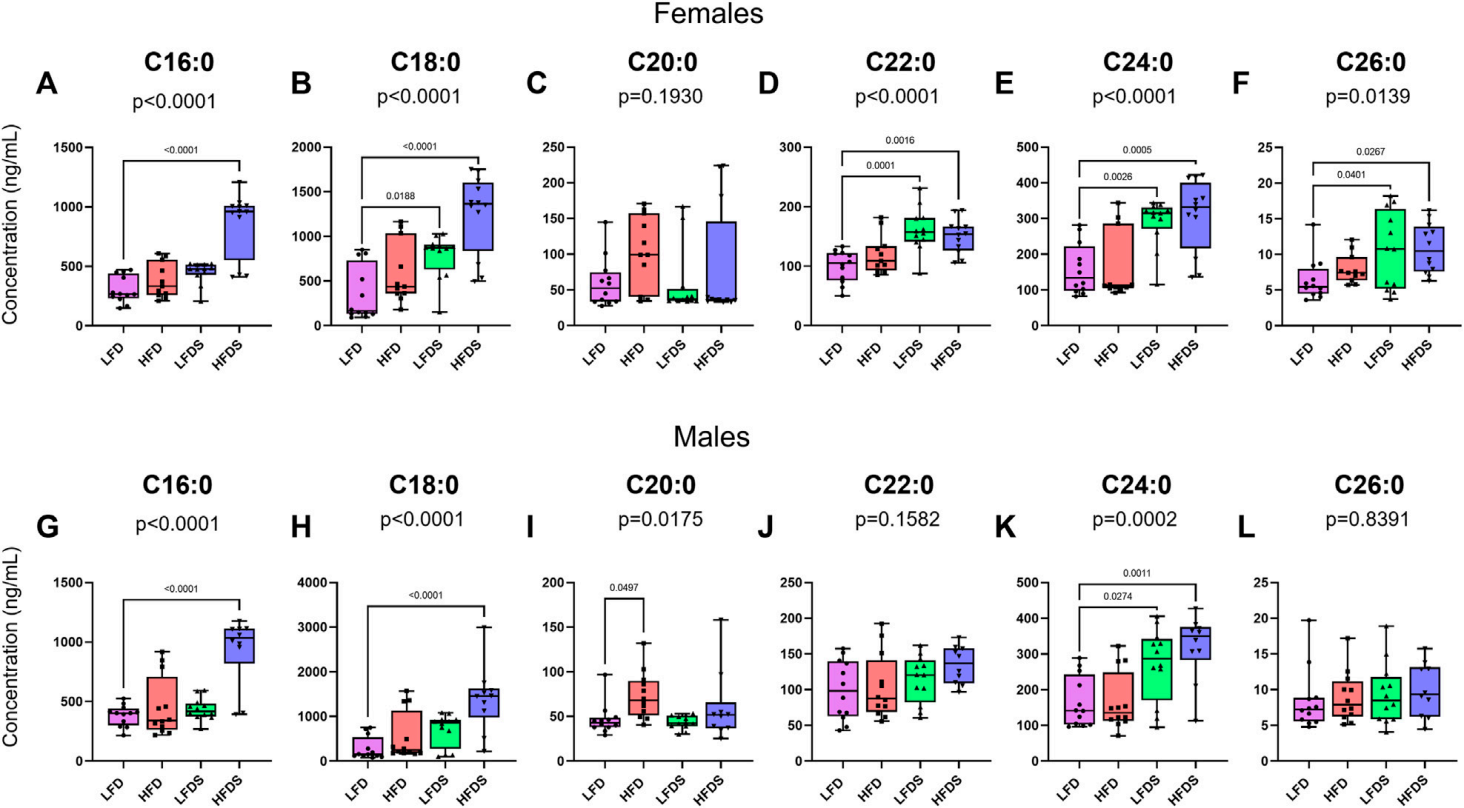
Ceramides in blood serum after HFD and/or STU stress. **(A–F)** Long-chain ceramides in blood serum from female rat groups. **(G–L)** Long-chain ceramides in serum of male rats. Data represented as mean ± SEM. Female sample sizes: LFD = 12, HFD = 11, LFDS = 12, and HFDS = 12. Male sample sizes: LFD = 12, HFD = 12, LFDS = 12, and HFDS = 10. Statistic used, One-way ANOVA with Tukey’s multiple comparison test.

Next, we assessed whether HFD or STU stress modified serum levels of long-chain ceramides in female rats (Figures 3A–F). At the end of behavioral testing total lipids were extracted from serum for determination of ceramide content. Despite the limited effects on behavior, HFD and/or STU stress induced many changes in serum long-chain ceramides. The female groups showed differences in the concentrations of C16:0 [F (3, 43) = 24.66, *p* < 0.0001], C18:0 [F (3, 43) = 14.85, *p* < 0.0001], C22:0 [F (3, 43) = 9.521, *p* < 0.0001], C24:0 [F (3, 43) = 10.52, *p* < 0.0001] and C26:0 ceramides [F (3, 43) = 3.968, *p* = 0.00139]. The female groups did not show differences in C20:0 ceramide [F (3, 43) = 1.645, *p* = 0.1930]. In females, adding STU stress to the LFD was associated with increased plasma levels of C18:0 (*p* = 0.0188), C22:0 (*p* = 0.0001), C24:0 (*p* = 0.0026) and C26:0 ceramides (*p* = 0.0401). Although switching females to a HFD did not cause changes in ceramide levels, adding STU stress with the HFD induced a synergistic increase in C16:0 (*p* < 0.0001) and C18:0 ceramides (*p* < 0.00084). Combining HFD and STU stress did not cause a further increase in C22:0 (*p* = 0.8485), C24:0 (*p* = 0.9439) or C26:0 ceramides (*p* = 0.9984) beyond the increase induced by STU stress alone. The combination of STU stress with HFD did not increase the levels of C20:0 ceramides (*p* = 0.7969).

Changes in ceramides were also observed in male rats (Figures 3G–L). The male groups showed differences in C16:0 [F (3, 42) = 17.67, *p* < 0.0001], C18:0 [F (3, 42) = 9.706, *p* < 0.0001], C20:0 [F (3, 42) = 3.769, *p* = 0.0175], and C24:0 ceramides [F (3, 42) = 8.545, *p* = 0.0002]. A HFD increased serum levels of C20:0 ceramides (*p* = 0.0497) compared to male rats fed a LFD. Exposing the LFD male group to STU stress promoted increased serum C24:0 ceramide (*p* = 0.0274). Combining STU stress in the setting of a HFD was associated with a synergistic increase in C16:0 (*p* < 0.0001) and C18:0 ceramides (*p* < 0.0001). Combing the STU stress and HFD did not further increase the C24:0 ceramides compared to the STU stress alone group (*p* = 0.5776) but the levels remained higher than the HFD group (*p* = 0.0011).

**FIGURE 4:**
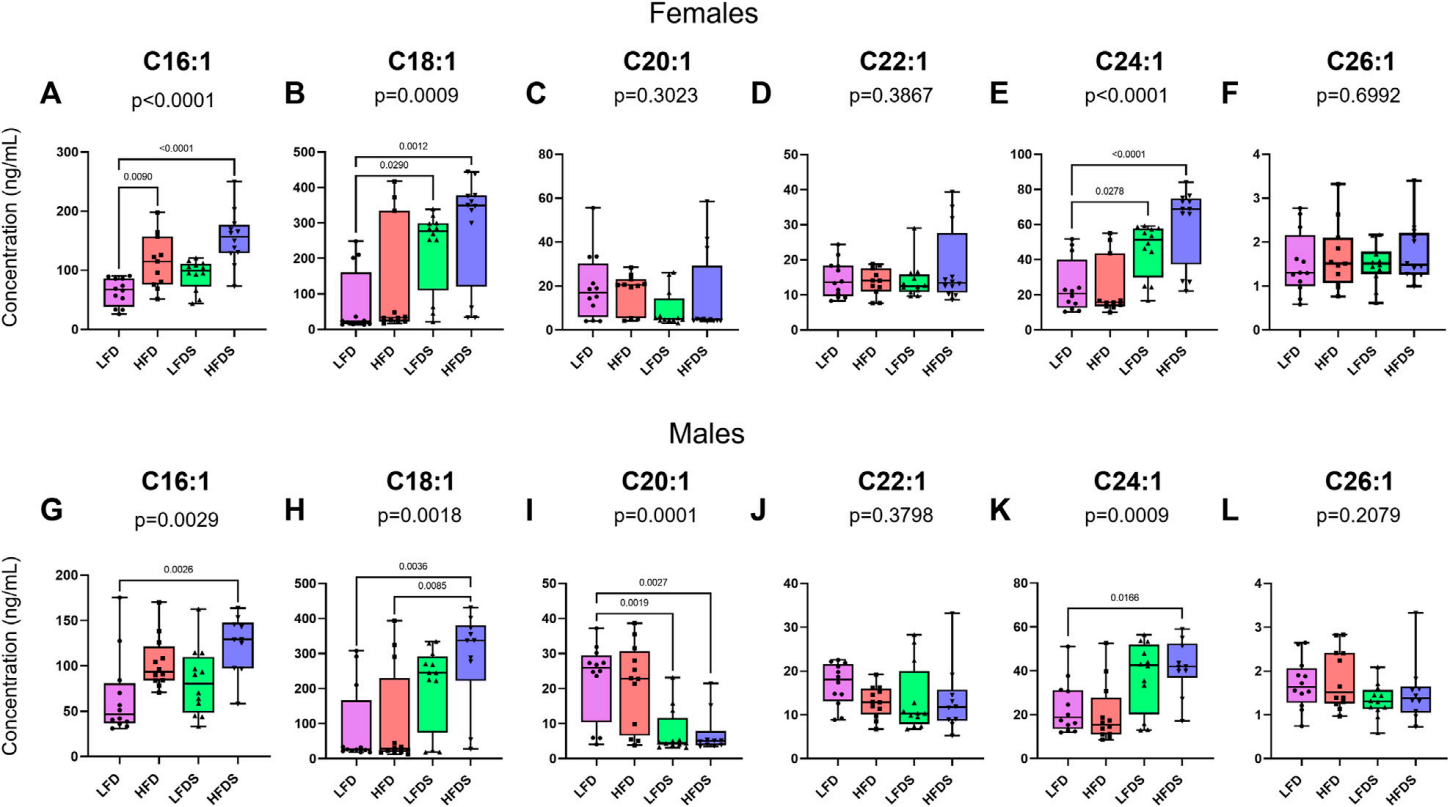
Ceramides with one unsaturated acyl chain in blood serum after HFD and/or STU stress. **(A–F)** Long-chain ceramides in blood serum from female rat groups. **(G–L)** Long-chain ceramides in serum of male rats. Data represented as mean ± SEM. Female sample sizes: LFD = 12, HFD = 11, LFDS = 12, and HFDS = 12. Male sample sizes: LFD = 12, HFD = 12, LFDS = 12, and HFDS = 10. Statistic used, One-way ANOVA with Tukey’s multiple comparison test.

To further examine the effects of HFD and/or STU stress on long-chain ceramides, we measured ceramides with one unsaturated acyl chain and found differences in the serum of both female and male rats (Figure 4). Female rats showed differences in the concentrations of C16:1 [Figure 4A, F (3, 43) = 13.53, *p* < 0.0001], C18:1 [Figure 4B, F (3, 43) = 6.570, *p* = 0.0009], and C24:1 [Figure 4E, F (3, 43) = 12.13, *p* < 0.0001], while no differences were found in the other ceramides evaluated. The inclusion of STU stress with LFD increased C18:1 (*p* = 0.0290) and C24:1 ceramides (*p* = 0.0278). In females, HFD alone increased C16:1 (*p* = 0.0090) and adding STU stress with HFD caused a further increase in C16:1 (*p* = 0.0477. Lastly, adding STU stress to HFD-treated female rats further increased the serum concentration of C18:1 (*p* = 0.0012) and C24:1 ceramides (*p* < 0.0001) compared to the STU stress alone group.

We also evaluated ceramides with one unsaturated acyl chain in serum from the male rats (Figures 4G–L). Male rats showed changing levels of C16:1 [F (3.42) = 5.481, *p* = 0.0029], C18:1 [F (3.42) = 5.959, *p* = 0.0018], C20:1 [F (3.42) = 8.846, *p* = 0.0001] and C24:1 ceramides [F (3.42) = 6.599, *p* = 0.0009]. Exposition to HFD alone was not enough to increase the levels of ceramides but adding the STU stress with the HFD caused a synergistic increase in C16:1 (*p* = 0.0026), C18:1 (*p* = 0.0036) and C24:1 ceramides (*p* = 0.0166). STU stress alone (*p* = 0.0019) or combined with HFD (*p* = 0.0027) was associated with reduced the levels of C20:1 ceramides. Overall, our findings suggests that both HFD and short-term stress contribute to selective increases in particular long-chain ceramides in the serum of female and male rats.

### High Fat Diet Combined With Short-Term Unpredictable Stress Altered TNF-α Expression in Female but Not Male Rats

**FIGURE 5:**
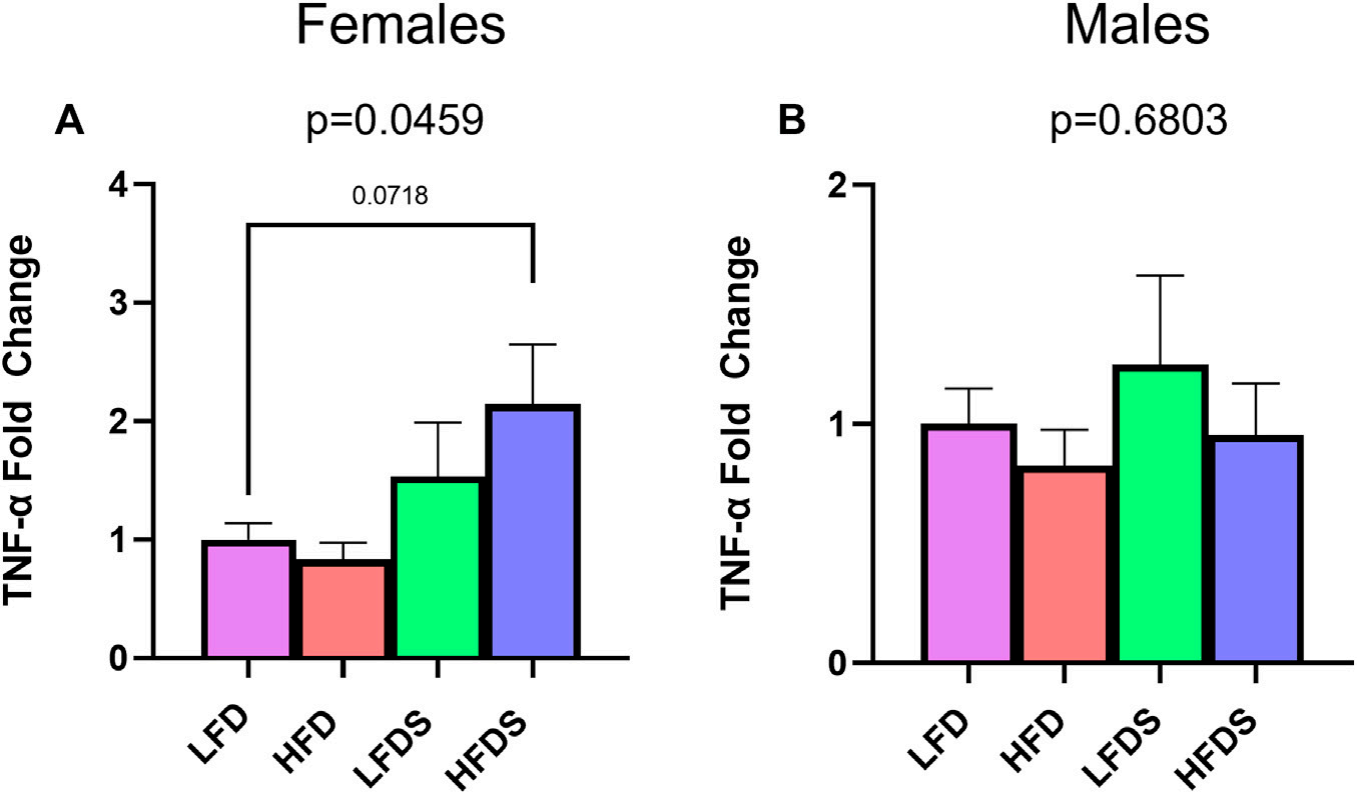
TNF-α in serum of female and male rats. **(A)** TNF-α expression in blood serum from female rat groups. **(B)** TNF-α expression in serum of male rats. Data represented as mean ± SEM. Female sample sizes: LFD = 12, HFD = 7, LFDS = 8, and HFDS = 9. Male sample sizes: LFD = 7, HFD = 9, LFDS = 10, and HFDS = 9. Statistic used, One-way ANOVA with Tukey’s multiple comparison test.

To examine further interactions of HFD and STU stress, we evaluated blood protein levels of the inflammatory cytokines, TNF-α and IL-6, which are associated with depressive-like behaviors (Haack et al., 1999; Berk et al., 2000; Liu et al., 2012; Dahl et al., 2014; Fan et al., 2017). We found a significant difference in TNF-α expression in the female groups [Figure 5A, F (3.32) = 2.981, *p* = 0.0459]. The HFD alone did not affect the protein levels of TNF-α (*p* = 0.9851) but adding the STU stress to HFD animals caused a trend towards increased expression in female rats (*p* = 0.0718). Contrary to females, male groups showed no differences in the expression of TNF-α [Figure 5B, F (3, 31) = 0.5071, *p* = 0.6803]. Moreover, the expression of IL-6 was below detection levels for all female and male groups.

## Discussion

Increases in long-chain ceramides have been found in the serum of patients with depression (Gracia-Garcia et al., 2011; Brunkhorst-Kanaan et al., 2019; Xue et al., 2020), suggesting that ceramides could contribute to the development of depressive behaviors. Although HFD or chronic stress can associate with serum ceramide accumulation (Boini et al., 2010; Wang et al., 2021), it is unclear which long-chain ceramides are altered and whether HFD and short-term stress can interact to induce synergistic changes in serum ceramides. In our study, rats fed a HFD for 8–10 weeks showed increased serum C16:1 ceramide in females and increased C20:0 in the males. In addition, 4 days of unpredictable stress alone was sufficient to increase C18:0, C18:1, C22:0, C24:1, and C26:0 in females and C24:0 in males and decrease C20:1 in males. We also found evidence of synergy, since combining the STU stress and the HFD increased C16:0, C16:1, and C18:0 ceramides in both sexes and C18:1 and C24:1 in males. Despite the changes in long-chain ceramides, exposure to HFD, STU stress, or HFD with STU stress did not promote anhedonia-like behavior in either sex. Furthermore, most of the increases in long-chain ceramides were observed in the groups exposed to the STU stress while only the HFD alone group showed increased despair-like behavior. Our data suggest that the changes in serum long-chain ceramides associated with HFD and/or STU stress may not be related to depressive-like behaviors.

To determine whether HFD and brief stress exposure interact to produce synergistic effects on serum long-chain ceramides in male or female rats, we combined HFD exposure of duration that is near the threshold for producing behavioral despair alone with 4 days of unpredictable stress. In addition to inducing despair-like behavior, the 8–10 weeks of HFD alone was sufficient to increase C16:1 ceramides in the serum of female rats and the C20:0 ceramides in the males. Ten weeks of HFD may be when most of the long-chain ceramides begin to increase, since 13–16 weeks of HFD increased C16:0, C18:0, C20:0, C22:0, and C24:0 ceramides in plasma of male mice (Shah et al., 2008; Boini et al., 2010). These results suggest that increases in C16:1 and C20:0 long-chain ceramides in the blood may contribute to the development of despair-like behavior in rodents. However, the C16:1 ceramides were also increased in the groups exposed to both HFD and STU stress that did not show increased despair-like behaviors. Furthermore, combining HFD and short-term stress increased C16:0, C16:1, and C18:0 ceramides in both sexes and C18:1 and C24:1 in males suggesting synergy between diet and stress in the production of C16:0 ceramides without inducing depressive-like behaviors in either sex. Overall, our findings suggest that although HFD and STU stress produce selective increases in long-chain ceramides associated with depression in people, the increases in ceramides are insufficient to induce depressive-like behaviors in rodents and may be unrelated to the changes in the depressive-like behaviors.

Although 6 weeks of HFD can increase TNF-α in serum in male mice (de Aquino et al., 2019), we did not detect changes in TNF-α protein in the males after 8–10 weeks of HFD or 4 days of STU stress alone or combined and only a trend towards increased TNF-α in the females exposed to HFD and STU stress combined. Longer exposure to HFD (16–20 weeks) have consistently shown increased protein levels of TNF-α in plasma of adult male rodents (Guillemot-Legris et al., 2016; Almeida-Suhett et al., 2017), suggesting that more extended exposure to HFD causes more robust increases in peripheral inflammation. Moreover, a more prolonged 35-days exposure to unpredictable stress may be needed to increase concentrations of TNF-α in adult male rodents (Pesarico et al., 2016). The lack of peripheral inflammation in our model may explain the lack of effects on depressive-like behaviors. After 8–10 weeks of exposition to HFD, our animals did not show differences in the weight gain compared to LFD animals. This suggests that our model may represent a pre-obesity model, since 14–20 weeks of HFD does increase the weight gain of male rodents (Kang et al., 2016; Mardare et al., 2016; Zemdegs et al., 2016). Although male rats exposed to HFD with 3 weeks of chronic stress showed increased weight gain (Pan et al., 2016), our inclusion of 4 days of unpredictable stressors did not cause weight changes in our animals but did reduce the kilocalories consumed in both sexes. Since food is rewarding, the reduced caloric intake of our animals exposed to 4 days of unpredictable stress could suggest that the stress induced a lack of pleasure that was not captured by the sucrose grooming task.

Despite the evidence of synergy in the production of serum long-chain ceramides, we did not find evidence of synergistic effects of HFD and 4 days of unpredictable stress on depressive-like behaviors in male or female rats. Studies have found that HFD for a period from 12 to 16 weeks increases depressive-like behaviors, while 8 weeks of HFD does not in male rodents (Dutheil et al., 2016; Almeida-Suhett et al., 2017; Lam et al., 2021). We found that 8–10 weeks of HFD was sufficient to produce despair-like behavior but not anhedonia-like behavior in male and female rats. Consistent with the studies suggest that 7–10 days of unpredictable stress are needed to cause depressive-like behavior in male rodents (Zurita et al., 2000; Maslova et al., 2002; Fang et al., 2021), we found that 4 days of two mild stressors each day was insufficient to increase behavioral despair in either sex. Previous studies found a lack of synergy between the HFD and chronic stress on anhedonia-like behavior and behavioral despair in male rodents (Finger et al., 2011; Wang et al., 2014). Consistent with these studies, we did not find synergistic effects from combining the HFD and STU stress on either anhedonia-like or despair-like behavior. In fact, we found that adding the unpredictable stress with the HFD led to less despair-like behavior than the HFD alone suggesting that the brief stress may have reversed the behavioral effects of the HFD.

An important limitation of our study is that we did not measure ceramide levels in the brain. Therefore, the increases in serum long-chain ceramides in our HFD-STU stress model may not have produced depressive-like behavior because the ceramides did not increase in the brain. However, since peripheral administration of fluorescently-labeled ceramides can accumulate in different brain structures within 30 min (Zimmermann et al., 2001), the serum ceramides in our model likely entered the CNS but may have been insufficient to induce depressive-like behaviors. Although previous studies found that 4–5 weeks of chronic unpredictable stress increases total ceramide concentration in various brain structures in male rodents, they did not assess whether the increases in ceramides correlated with increases in depressive-like behaviors (Gulbins et al., 2013; Oliveira et al., 2016). However, direct infusion of C16:0 ceramides into the dorsal hippocampus of male mice induced anhedonia-like behavior (Gulbins et al., 2013) suggesting that if the long-chain ceramides reach a sufficient concentration in the brain they can induce depressive-like behavior.

In conclusion, our findings suggest that brief exposure to HFD or unpredictable stress is sufficient to increase the concentrations of certain long-chain ceramides, associated with depression in people, in the serum of male and female rats. Furthermore, exposure to HFD and unpredictable stress caused a synergistic increase in C16:0, C16:1, and C18:0 ceramides in both sexes and C18:1 and C24:1 in males. However, the increased peripheral long-chain ceramides were not associated with increases in depressive-like behaviors suggesting that increases in serum long-chain ceramides may not be associated with the development of depressive-like behaviors in rodents.

## Data Availability

The raw data supporting the conclusion of this article will be made available by the authors, without undue reservation.
